# Differentiated Thyroid Cancer—Treatment: State of the Art

**DOI:** 10.3390/ijms18061292

**Published:** 2017-06-17

**Authors:** Benedikt Schmidbauer, Karin Menhart, Dirk Hellwig, Jirka Grosse

**Affiliations:** Department of Nuclear Medicine, University of Regensburg, 93053 Regensburg, Germany; benedikt.schmidbauer@ukr.de (B.S.); karin.menhart@ukr.de (K.M.); dirk.hellwig@ukr.de (D.H.)

**Keywords:** differentiated thyroid cancer, radioiodine therapy, targeted therapy, tyrosine kinase inhibitors

## Abstract

Differentiated thyroid cancer (DTC) is a rare malignant disease, although its incidence has increased over the last few decades. It derives from follicular thyroid cells. Generally speaking, the prognosis is excellent. If treatment according to the current guidelines is given, cases of recurrence or persistence are rare. DTC requires special expertise by the treating physician. In recent years, new therapeutic options for these patients have become available. For this article we performed a systematic literature review with special focus on the guidelines of the American Thyroid Association, the European Association of Nuclear Medicine, and the German Society of Nuclear Medicine. For DTC, surgery and radioiodine therapy followed by levothyroxine substitution remain the established therapeutic procedures. Even metastasized tumors can be cured this way. However, in rare cases of radioiodine-refractory tumors, additional options are to be discussed. These include strict suppression of thyroid-stimulating hormone (also known as thyrotropin, TSH) and external local radiotherapy. Systemic cytostatic chemotherapy does not play a significant role. Recently, multikinase or tyrosine kinase inhibitors have been approved for the treatment of radioiodine-refractory DTC. Although a benefit for overall survival has not been shown yet, these new drugs can slow down tumor progression. However, they are frequently associated with severe side effects and should be reserved for patients with threatening symptoms only.

## 1. Introduction

Patients with differentiated thyroid carcinoma have an excellent prognosis. The multimodal therapeutic approach is risk-adapted to achieve optimal treatment of differentiated thyroid cancer (DTC) and to minimize treatment-related morbidity. The treatment includes surgery (near-/total thyroidectomy) usually followed by remnant ablation using radioiodine according to the guidelines of the American Thyroid Association (ATA) and European Association of Nuclear Medicine (EANM) as well as a risk-stratified follow-up including hormone substitution.

However, in patients with primary or secondary radioiodine-refractory thyroid carcinoma the prognosis becomes significantly poorer. External beam irradiation may be used for locoregional control. Receptor tyrosine kinase inhibitors (TKIs) have shown clinical effectiveness in iodine-refractory DTC.

In this review, we present the current state of treatment of DTC.

## 2. Epidemiology and Classification

DTC is a rare disease with mostly excellent prognosis. The appearance of DTC depends on age, sex, family history, radiation exposure and many other factors [1]. DTC occurs in 7–15% of patients with thyroid surgery. In the year 2014, approximately 63,000 new cases of DTC were diagnosed in the US [2] compared to 2009 with only 31,200 new cases. In Germany there are about 6000 new cases of DTC per year. The growing incidence of thyroid cancer and the tumor shift to diagnosis of smaller tumors is due to the increased usage of diagnostic methods, such as ultrasound of the neck [3].

Differentiated thyroid cancer includes papillary and follicular cancer that derive from thyrocytes and express the sodium iodine symporter. DTC represents the majority (90%) of all types of thyroid cancer [4]. One study predicts that papillary thyroid cancer will become the third most expensive cancer in women, with costs of US$ 19–21 billion in the US in 2019 [5].

Worldwide, there are many clinical practice guidelines for diagnosis, therapy and follow-up of DTC. The European Thyroid Association (ETA) published new guidelines for the management of DTC in 2013 [6]. The Society for Nuclear Medicine and Molecular Imaging and European Association of Nuclear Medicine published their most recent guidelines for radioiodine therapy of differentiated thyroid cancer in 2012 and 2008, respectively [7,8]. The Japanese Association of Endocrine Surgeons and the Japanese Society of Thyroid Surgeons recently reviewed their guidelines in 2014 [9]. The new ATA guidelines for management of differentiated thyroid cancer for adults were published in 2015 [10]. The updated ATA guidelines for management of DTC for children were also published in 2015 [11].

The risk classification of DTC using multiple staging systems is based on a combination of the size of the primary tumor, specific histology, extrathyroidal spread of the tumor and the age at diagnosis. It helps to predict the risk of local recurrence and developing metastases and the mortality in patients with DTC. The TNM classification depends on the size of primary tumor, the number and localization of metastatic lymph nodes and number of distant metastases (Table 1) [12]. The American Joint Committee on Cancer (AJCC) uses the combination of TNM Classification and an age of more than 55 years at diagnosis as risk factor [13]. The differentiation of lymphatic invasion and angioinvasion is of high importance, because angioinvasion is associated with an intermediate risk of recurrence. A common risk-stratification of DTC is based on the TNM classification (see also Section 4.2) [14]:

- high-risk group: pT3, pT4, each N1, all M1;
- low-risk group: pT1b, pT2, cN0/pN0, cM0;
- very low risk-group: pT1a, cN0/pN0, cM0.

The American Thyroid Association defines in their current guideline a stratification based on the risk of structural disease recurrence [10]:

- high-risk group: gross extrathyreoidal extension, incomplete tumor resection, distant metastases, or lymph node >3 cm;
- intermediate-risk: aggressive histology, minor extrathyreoidal extension, vascular invasion, or >5 involved lymph nodes (0.2–3 cm);
- low-risk: intrathyreoidal DTC, ≤5 lymph nodes micrometastases (<0.2 cm).

In the last few years new molecular and genetic biomarkers, such as BRAF (V600E), phosphatidylinositol 4,5-bisphosphate 3-kinase catalytic subunit α (PIK3CA), tumor protein p53 (TP53), RAC-α serine/threonine-protein kinase 1 (AKT1) and telomerase reverse transcriptase (TERT) became more important for the management of diagnosis, therapy and observing of DTC. The role of RAS is discussed controversally. Table 2 shows the impact of the two well-evaluated molecular markers BRAF and TERT [15]. Some of these alterations might be interesting molecular targets for new therapies.

### 2.1. Papillary Thyroid Cancer

Papillary thyroid carcinoma (PTC) is the most common form of DTC. Histologically it is a tumor of follicular cells of the thyroid gland with characteristic nuclear signs. There are more than 10 histological variants of papillary thyroid cancer documented, can be seen in Table 3 [16,17]. Due to this microscopic diversity, different risk stratifications are needed.

The tall cell variant is one of the tumor entities with unfavorable outcome. This type of thyroid cancer is presented in tall columnar cells and occurs in older age showing a higher rate of lymph node metastases. In nearly 80% of these tumors the BRAF (V600E) mutation is found [18]. A new aggressive variant of papillary thyroid carcinoma, which is characterized by cells with hobnail appearance and apically placed nuclei, was described recently. The BRAF (V600E) mutation is found frequently and associated with distant metastases [19]. In children and adults affected by the Chernobyl incident the solid variant of PTC appears predominantly. Mortality within the first 10 years after initial diagnosis and treatment is low (<1%) [20,21]. It is very important to recognize that there are histological differences compared to poorly differentiated carcinomas, because of the very different therapy strategy. In poorly differentiated thyroid cancer the capability to take up (radio) iodine is clearly reduced (e.g., decreased expression of sodium iodine symporter) and therefore not sufficient to achieve a significant therapeutic effect. Another form of PTC is the diffuse sclerosing variant. It is characterized by a higher incidence of lymph node and distant metastases. Nevertheless, overall mortality appears low. The encapsulated follicular variant of papillary carcinoma very rarely shows capsular or vascular invasion. Histologically it is characterized by follicular growth, typical nuclear features of papillary carcinoma and total tumor encapsulation. RAS mutations can be detected frequently. The non-encapsulated follicular variant of papillary cancer shows BRAF (V600E) mutations quite often [22,23]. This tumor is associated with lymph node metastases in about 25–30% and low rates of distant metastases.

PTC presents distant metastases mainly in bones or lungs.

Papillary microcarcinoma is a PTC < 1 cm corresponding to the classification of the World Health Organization (WHO) which is often found incidentally. In some autopsy studies the papillary microcarcinoma was found in 6–35% of the thyroids by incident [10]. Papillary microcarcinoma may also exhibit RET proto-oncogene (RET)/PTC-rearrangements or BRAF (V600E) mutations.

### 2.2. Follicular Thyroid Cancer

Follicular thyroid carcinoma (FTC) is a malignant tumor, histologically derived from follicular thyroid cells, showing transcapsular or vascular invasion and missing the typical nuclear signs of papillary carcinoma. In the traditional classification of FTC there are two groups: minimally invasive and widely invasive [24,25,26]. The widely invasive FTC shows an extensive vascular invasion, often also associated with extrathyroidal growth.

Oncocytic follicular carcinoma is a special form of FTC with some microscopic differences compared to conventional FTC. One of them is the accumulation of innumerable mitochondria. Due to its histological differences, oncocytic carcinoma shows some different biological behavior with a higher ability to metastasize to lymph nodes and a possibly higher rate of recurrence and tumor-related mortality [27,28,29].

### 2.3. Familial Tumor Syndromes

Some of the histopathological variants of DTC are associated with familial tumor syndromes. For example, the cribriform-morular form of papillary thyroid cancer is frequently seen in patients with a germline mutation in adenomatous polyposis coli gene [30,31]. About 40% of patients with this special histological form of papillary thyroid carcinoma show simultaneously a familial adenomatous polyposis (FAP) [32]. Due to this high rate of association of cribriform-morular PTC and FAP it is very important to complete the diagnostic work-up with colonoscopy and genetic counseling.

Another type of FTC is associated with the germline mutation of the phosphatase and tensin homolog (PTEN) gene [33,34,35]. The follicular variant of thyroid carcinoma is in this case very characteristic and should be known by pathologists. The syndrome is associated with a high risk of appearance of other tumors, such as colon hamartomas or breast and endometrium tumors. Genetic counseling is recommended.

## 3. Diagnostic Approach to Thyroid Nodules

The prevalence of sonographically-detected thyroid nodules in the U.S. is described between 19% and 35% [36]. Toxic adenomas are found in up to 4 percent of the population. In Europe the incidence of thyroid nodules is higher in some areas. In Germany, a country with relative iodine deficiency, nodules are found in 33% of the population.

Risk factors for malignancy are exposure to ionizing radiation through radiotherapy or fallout especially in younger years, familial thyroid carcinoma, or syndromes that are associated with thyroid cancer like PTEN, Cowdens disease or multiple endocrine neoplasia type 2 (MEN2). Warning signs in clinical examination are rapid nodule growth, fixation in the surrounding tissue, vocal cord paralysis, possibly accompanied by hoarseness.

The diagnostic cornerstone of thyroid nodules remains the ultrasound examination. It should be performed in any case of known or suspected thyroid nodules or cervical lymphadenopathy to assess if further diagnostic is needed. Sonographic patterns suspicious of malignancy are microcalcifications, irregular margins, solid consistency, hypoechogenity, extrathyroidal extension (ETE) and a tall shape rather than a wide one. Intranodulary vascularization does not seem to have a clear correlation with malignancy [10].

**Figure 1:**
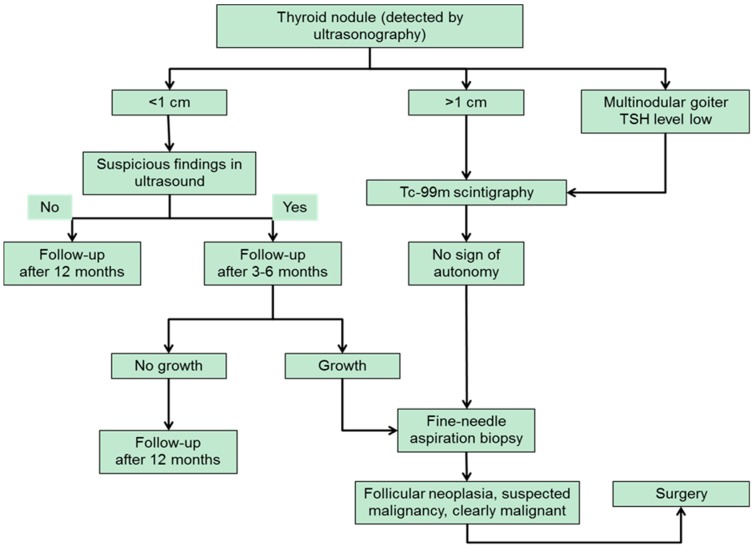
Diagnostic algorithm for the evaluation of thyroid nodules (modified from [38]). TSH: thyroid-stimulating hormone.

Roughly one third of thyroid nodules are larger than 1 cm and eligible for scintigraphy [37]. The guidelines of the German Society of Nuclear Medicine recommend a scintigraphic examination of every thyroid nodule >1 cm. By routinely performing a Tc-99m thyroid scan autonomous adenomas that have not yet an impact on thyroid-stimulating hormone (TSH) level can be detected without subjecting the patient to the risks and stress of fine-needle aspiration (FNA). This applies especially to groups at increased risk for complications like patients that are treated with coagulation inhibitors. The diagnostic algorithm for evaluation of thyroid nodules according to the German guidelines that was recently published by Feldkamp et al. is shown in Figure 1 [38].

The ATA guidelines recommend measurement of the TSH level if a thyroid nodule is found. A radionuclide scan (Tc-99m, preferable I-123) should be performed only if TSH level is subnormal [10]. Nevertheless, the American guidelines cannot be applied to the rest of the world without adjustment for differences in the patient populations. Except for clearly benign cysts, for every lesion of a certain size ultrasound guided fine-needle aspiration biopsy (FNA) is recommended (Table 4) [10]. Furthermore, the measurement of serum calcitonin is recommended when new thyroid nodules are detected time to rule out medullary thyroid cancer that derives from c-cells and is not added to group of DTC.

Cytological analysis is performed according to the Bethesda System for Reporting Thyroid Cytopathology. The findings are graded into six categories:

I: nondiagnostic/unsatisfactory;
II: benign;
III: atypia of undetermined significance/follicular lesion of undetermined significance;
IV: follicular neoplasm/suspicious for follicular neoplasm;
V: suspicious for malignancy;
VI: malignant.

If the FNA biopsy is graded non-diagnostic/unsatisfactory, biopsy should be repeated. Numerous molecular tests can be applied to distinguish malignant from benign lesions, such as BRAF (V600E), PIK3CA and TERT promoter, AKT1, and TP53, although there is no explicit recommendation in the current guidelines. Accordingly, adjustments are to be expected in the future [15]. For more non-diagnostic biopsies in a row, the decision for close surveillance without intervention or for surgery should be made in dependence of the sonographic pattern [10].

## 4. Therapy of Differentiated Thyroid Carcinoma

DTC should be treated interdisciplinary in facilities with an appropriate expertise in order to ensure an optimal long-term treatment quality. Specialists in surgery, endocrinology, pathology and nuclear medicine should be available. The therapeutic approach is individualized and risk-adapted.

### 4.1. Surgery

For widely invasive follicular thyroid carcinomas and FTC with vascular infiltration, thyreoidectomy is recommended. Lymph node dissection is recommended if lymph node metastases can be detected pre- or intraoperatively by sonographic examination and/or palpation. The solitary minimally invasive FTC without vascular invasion does not require a second surgical intervention as completion, if the tumor has been completely removed (R0). Thyreoidectomy and lymph node dissection of the central compartment are recommended for prognostically unfavorable variants.

For all papillary thyroid carcinomas >1 cm and/or for all metastasized or macroscopically invasive PTC irrespective of size, thyreoidectomy is recommended [10,39]. If lymph node metastases have been detected sonographically or intraoperatively, lymph node dissection in the affected compartment should be done to reduce the risk of (local) recurrence. On the other hand, the diagnostic or therapeutic extirpation of only single lymph nodes as a part of the primary intervention is not recommended. Although at present the importance of central lymph dissection with prophylactic intention is still unclear, the high probability of lymph node metastases is a substantial argument to expand the surgical procedure. Furthermore, it is difficult to exclude lymph node metastases pre- or intraoperatively. After all, the increased risk of a local recurrence associated with an increased morbidity due to the surgical intervention in the situation of relapse should be mentioned. On the other hand, the main arguments against a prophylactic dissection are the lack of evidence regarding a better outcome of the patients and the remarkably higher complication rate due to the more extensive intervention (e.g., vocal cord paralysis, parathyreoprival tetany). Specifically, papillary thyroid microcarcinoma that is found incidentally does not require a further surgical treatment.

After all, accurate histopathological examination of the specimen after (hemi)thyroidectomy and lymphadenectomy (if done) is regarded as the gold standard and is indispensable for the management and further diagnostic and therapeutic approach.

### 4.2. Adjuvant Radioiodine Therapy

Radioiodine therapy (RIT) has been established for more than 60 years. The benefit was demonstrated in DTC patients with a high risk for recurrence. In patients with very low-risk DTC a positive effect of a RIT on tumor-free and overall survival has not been proven by prospective clinical trials.

RIT is defined as the systemic administration of I-131 (radioiodine as sodium iodide or potassium iodide) to irradiate thyroid remnants as well as non-resectable or incompletely resected DTC.

Adjuvant ablative RIT of thyroid remnants or tumor tissue is the optimal precondition for the follow-up including determination of serum thyroglobulin (Tg) and I-131 whole-body scans. The rationale that underlies this approach is to detect a local recurrence or distant metastases in an early and potentially curable stage to minimize mortality. However, regional or distant metastases frequently are only detectable by rising Tg levels after a successful remnant-ablation. It was shown that an ablative RIT decreases the rate of recurrence and mortality over a follow-up period of more than 10 years [40,41,42,43]. RIT is indicated in high-risk DTC (pT3, pT4, each N1, every M1), in low-risk DTC (pT1b, pT2, cN0, pN0, M0) and in small papillary thyroid carcinoma (very low-risk DTC), if there are risk factors (see Section 4.7) [44,45]. Furthermore, RIT can be used for the treatment of radioiodine-positive tumor residues, lymph node and distant metastases with curative or palliative intention. In the case of tumor activity shown by an increasing serum level of thyroglobulin without a macroscopically detectable tumor using morphological and functional imaging RIT can be carried out after carefully weighing risks and benefits [14].

To ensure a high uptake of radioiodine (I-131) in remnant tissue, (suspected) tumor, or metastases, an elevated serum level of TSH is required (>30 mU/L). This level is believed to increase the expression of the sodium iodine symporter (NIS) in benign and malignant follicular cells of the thyroid [46]. According to the guidelines of the ATA and EANM [8,10] this TSH level can be reached by waiting not less than 3 weeks after thyroidectomy or after a withdrawal (4–5 weeks) of levothyroxine (LT4). The subsequent period of hypothyroidism decreases the quality of life significantly in many patients. The physical and psychological symptoms of hypothyroidism include gain of weight, impaired renal function, cardiovascular abnormalities, dyslipidemia (exacerbation), constipation, dry skin, hoarseness, fatigue, sleep disturbance, impaired ability to concentrate and depression [47]. Alternatively, recombinant TSH (rhTSH) can be administered intramuscularly (2 times 0.9 mg rhTSH) to avoid inconvenience and morbidity due to the lack of thyroid hormone. This drug is approved for radioiodine ablation (without known distant metastases) of T1–4 tumors, diagnostic whole-body scan and preparation for testing of serum Tg in adults [48,49,50,51].

Absolute contraindications of RIT are pregnancy and breastfeeding. Relative contraindications include depression of the bone marrow (especially if the administration of high activities of I-131 is planned), a restriction of salivary gland function, pulmonary function restriction (if a high accumulation of I-131 in lung metastases is possible) and symptomatic metastases of the central nervous system, because local edema and inflammation caused by RIT and hypothyroidism can lead to severe compression effects [8].

The activity of I-131 for remnant ablation is still discussed controversially. The HiLo study (Great Britain) and the ESTIMABL study (France) both compared ablative RIT with 1.1 GBq I-131 versus administration of 3.7 GBq (100 mCi) I-131 after thyroid hormone withdrawal or stimulation with rhTSH in patients with low-risk carcinoma [52,53]. Both studies showed that RIT with only 1.1 GBq (30 mCi) I-131 is not inferior compared to the higher activity in regard to the success of ablation. However, the definition of “success of ablation” used in both studies is not accepted by all departments and associations. Several authors report that the rate of a second RIT increases when low therapy activities were used initially [54,55].

In the ESTIMABL study the diagnostic I-131-whole-body scan 8 months after I-131 ablation was limited to patients with elevated Tg antibodies and disturbed Tg recovery. Even in this subgroup this concept was not consistently implemented. Based on an observation study, iodine-accumulating metastases are possible with a measurable Tg level of up to 1 ng/mL [56]. Due to the open question of the “optimal” activity for remnant ablation, the German Society of Nuclear Medicine for example recommends a single administration of 1 to 3.7 GBq (about 30–100 mCi) I-131 [14]. Preablation scanning with Tc-99m pertechnetate on the day of ablation (as used in the HiLo trial [52]) can give very useful information in clinical decision making. In low-risk DTC patients with a large remnant (multiple foci or one large focus) ablation with 3.7 GBq (30 mCi) may be prefered.

Although RIT is generally well tolerated, the procedure has some potential short- and long-term side effects [8]. Short-term risks/side effects are: thyroiditis due to irradiation, swelling of the tumor or metastases (including compression symptoms), gastritis and nausea, sialadenitis and abnormalities of taste and smell, bone marrow depression, and hypospermia.

Long-term risks and side effects include permanent bone marrow depression, second primary malignancy after RIT with a high cumulative activity (leukemia and solid tumors) [57], chronic sialadenitis (including abnormalities of taste and smell, xerostomia,) and pulmonary fibrosis (in patients with diffuse iodine-avid pulmonary metastases). Due to the risk of chronic hypospermia or azoospermia, sperm banking should be considered if high cumulative activities are expected [58]. These risks have to be weighed against the expected benefits of the RIT.

### 4.3. Metastatic Differentiated Thyroid Carcinoma

Distant metastases occur in patients with differentiated thyroid carcinoma with a prevalence of up to 10%. In particular, they affect lung and bone [59]. If a sufficient uptake of I-131 in metastases is measurable, different therapeutic approaches are to be weighed regarding risk and benefit.

If locoregional lymph node metastases are detectable, surgery should be performed. I-131 is used for iodine-avid metastases for treatment control after surgery or as an alternative therapy if no surgery is possible/planned (e.g., additional detection of distant metastases requiring RIT, previously performed radiotherapy, previous lymph node dissection).

In the case of micronodulary metastases of the lung RIT is carried out as a treatment with curative intent. Macronodulary pulmonary metastases should also be treated with I-131 in a curative intention but a complete remission is unlikely. Alternatively, (or in combination) the resectability can be evaluated.

The complete surgical resection of isolated bone metastases leads to an improved outcome. A combination of different therapeutic approaches like percutaneous radiotherapy, RIT and local interventional therapy could be helpful if symptomatic metastases of the bone cannot be (completely) resected. The same strategy is applied to brain metastases [14].

For the treatment of metastases, standard activities of 4–11 GBq (about 100–300 mCi) I-131 are given depending on individual patient characteristics like age, renal function, bone marrow depression and tumor load.

### 4.4. Thyroid Hormone Treatment

After thyroidectomy, life-long thyroid hormone therapy is required, usually as monotherapy with levothyroxine (LT4). Since TSH is able to promote the growth of remaining DTC cells, the dosage of LT4 should initially be high enough to achieve a suppression of thyrotropin. The thyroid function should be checked after 6 to 8 weeks. Depending on the result the dosage should be adjusted. An elevated level of triiodothyronine has to be avoided.

A long-term suppression of TSH to values <0.1 mU/L is currently only recommended for high-risk patients and patients with persistent disease indefinitely in the absence of specific contraindications [10]. In these cases, a better prognosis was demonstrated for the suppression of thyrotropin. No evidence-based data are available for optimal duration of TSH suppression.

According to the guidelines of the ATA, serum TSH should be maintained between 0.1 and 0.5 mU/L in patients with high-risk disease but excellent or intermediate response to therapy for up to 5 years and also in patients with a biochemical incomplete response taking into account the initial ATA risk classification published by Haugen et al. [10]. This recommendation is rated as weak with low-quality evidence. If the response to therapy is excellent biochemically and clinically in patients with a low risk for recurrence and there is no evidence of disease in the course of time, the serum level of TSH may be kept in a range of 0.5–2.0 mU/L, because there is no data showing a benefit of TSH suppression for low-risk patients.

Individual patient-related factors such as osteoporosis or osteopenia and cardiac co-morbidities like atrial fibrillation should always be taken into account during thyroid hormone therapy and weighed against the risk of recurrence. Especially in elderly patients >60 years, the use of TSH suppressive therapy should be carefully considered since the risk of such complications is significantly increased [60].

### 4.5. Follow-Up

Although the cumulative relapse rate is up to 30%, the life expectancy of DTC patients (pT1-3, pN0-1, M0) is not significantly different from the general population after therapy according to the current guidelines. Lifelong follow-up examinations should be carried out because relapses can occur even after decades and may be cured again. Initial checks should be carried out every six months (e.g., for the first 5 years after diagnosis). If there are no pathological findings later on, annual examinations are adequate [61]. The follow-up examination is based on the medical interview, clinical examination, cervical sonography, determination of TSH, triiodothyronine, levothyroxine, and thyroglobulin including Tg antibodies. In the case of postoperative hypoparathyroidism, the substitution therapy (cholecalciferol, calcium) should be checked and adapted (if necessary) to minimize the risk of osteoporosis.

A diagnostic whole-body scan is obligatory 6–12 months after initial RIT, a second scan is only needed in the case of relapse [10,62].

The criteria for a disease-free stage 6–12 months after primary therapy of DTC with total thyroidectomy ± radioiodine therapy are no clinical signs of DTC, no pathological uptake in the I-131 whole-body scan (only after remnant ablation) and a serum Tg below the detection limit (under suppression and after TSH stimulation, with absence of Tg antibodies) [10,62,63]. Under these conditions patients have a very low probability of relapse. If there are signs of relapse (e.g., elevated/rising serum levels of Tg) and no radioiodine-accumulating tumor tissue is detectable, clinical diagnostics should include the search for non-radioiodine-avid tumor tissue using F-18-fluorodeoxy-glucose positron emission tomography (FDG-PET) combined with computed tomography ideally under TSH-stimulation.

### 4.6. Tyrosine Kinase Inhibitors

For poorly differentiated thyroid carcinoma without relevant iodine metabolism and therefore very low radioiodine uptake, RIT is not a therapeutic option. Radioiodine resistance is currently defined as lesions without iodine uptake under TSH stimulation, progression in size in the year following RIT or persistent metastases after a cumulative dose of 22 GBq (600 mCi) I-131 of radioiodine. In these cases, complimentary diagnostic using FDG-PET/computed tomography (CT) is essential. FDG uptake is typically increased in poorly differentiated lesions that can be overlooked on radioiodine scans. Prognosis for radioiodine resistant thyroid cancer with distant metastases is very poor, with an estimated median survival time of about 2.5 to 3.5 years [64,65].

Chemotherapy comes at high toxicity with disappointing response rates [66]. For these patients a strict LT4 regime with TSH suppression is the best way to go. On showing rapid progression under such a regime, therapy options were few until recently.

Tyrosine kinase inhibitors like vandetanib, sorafenib and lenvatinib are a relatively new approach to systemic therapy in these cases. Tyrosine kinase receptors, the target structure of TKI, are trans-membrane proteins that mediate cell survival and proliferation [67]. If mutated, they can cause uncontrolled cell proliferation, dedifferentiation and apoptosis reduction. A large part of DTC show at least one mutation of RAF, RET or paired box gene 8 (PAX8)/peroxisome proliferator-activated receptor gamma (PPARγ) which makes them targets for TKI therapy. Furthermore, TKIs block receptors of the vascular endothelial growth factor (VEGF), fibroblast growth factor receptors and platelet-derived growth factor and thus inhibit tumor angiogenesis and lymphangiogenesis and cause hypoxia in malignant tissue [68]. TKIs, already approved for the treatment of irresectable liver cancer and renal carcinoma, promise to be an effective new tool for the treatment of poorly differentiated thyroid carcinoma (PDTC) [66].

A recent review on the use of sorafenib, sunitinib and lenvatinib showed a benefit for progression-free survival of up to five months [69]. While initially showing partial response or at least disease stabilization after sorafenib, the first TKI approved for thyroid cancer, patients almost always develop resistance over the course of the following one to two years. Switching to another TKI is possible at this point [70]. A study using lenvatinib was able to indicate prolonged progression-free survival regardless of BRAF or RAS mutation status, suggesting a diminished role of these pathways [71]. However, a benefit in overall survival could not be found.

Therapy with kinase inhibitors may be accompanied by severe side effects. Induced hypertension is one of the most common; the underlying pathophysiology is yet unclear. Vasoconstriction following reduced nitric oxide production via inhibition of the VEGF-PI3K pathway is discussed. A reduction of peripheral arterioles due to antiangiogenic effects resulting in increased peripheral resistance and an activation of the endothelin-1-system causing vasoconstriction have also been suggested [72]. Other side effects may include diarrhea, fatigue, hepatotoxicity, skin changes, nausea, increased LT4 dosage requirement, changes in taste and weight loss and associated with a severe decrease of quality of life [69].

Keeping this in mind kinase inhibitors can certainly not be considered as a standard regime or an alternative to TSH-suppression. For patients with radioiodine-refractory DTC they can be a useful complementation to standard therapy.

While undergoing TKI therapy patients can display somewhat inconclusive lab results. Normally a reliable parameter, thyroglobulin levels can fluctuate under TKI treatment. These changes do not necessarily represent the actual course of the disease as it is monitored in anatomical imaging. Sufficient therapeutic monitoring not only by relying on lab tests but also on CT or PET/CT diagnosis to determine a morphologic or metabolic response is essential [73].

Considering the extensive side effects, this therapy should be reserved for patients with rapid tumor progression and severe to life threatening symptoms. In these cases, the decision for TKI therapy should be made in a interdisciplinary manner, carefully weighed against local strategies like radiotherapy and local surgery [74]. TKI treatment should only be performed by a team of physicians experienced with side effects management.

The European guidelines for treating differentiated thyroid carcinoma are from 2008. Kinase inhibitors are therefore not considered there [8] and a unanimous European recommendation is still awaited.

### 4.7. Papillary Microcarcinoma

Because of the excellent prognosis of papillary microcarcinoma (PTMC), a hemithyroidectomy without RIT is regarded as sufficient therapy, if there is no sign of local invasion, lymph node and/or distant metastases. The substitution of LT4 should keep the serum level of TSH in a euthyroid metabolic state.

In a meta-analysis, PTMC showed a prevalence of distant metastases of 0.4%, a probability of locoregional relapse of 2.5% but also a prevalence of micrometastases in locoregional lymph nodes of 12–50% [45]. The risk of lymphogenic micrometastases increases with increasing tumor diameter [75]. Using single photon emission computed tomography (SPECT) combined with CT, other studies showed a prevalence of lymph node metastases up to 57% [76,77]. The relapse-free survival in patients with PTMC after 5 years was 78.6% without RIT compared to 95.0% in patients that have had a remnant ablation with RIT [78]. The recommendation for a RIT in PTMC is based on the extent of resection and the individual risk profile. Risk factors are multifocality, infiltration of the thyroid gland, histological variants of papillary thyroid carcinoma, low degree of differentiation, tumor diameter 6–10 mm, molecular markers like BRAF-V600E mutation, infiltrative tumor growth, surrounding desmoplastic fibrosis and previous percutaneous irradiation of the neck [14]. In patients with a residual thyroid gland (e.g., after lobectomy) an ablative RIT is not indicated.

## 5. Summary and Conclusion

Differentiated thyroid cancer is a rare tumor entity but shows a strongly increasing incidence over the last decades. It derives from the follicular epithelium of the thyroid and shows basic biological characteristics of healthy thyroid tissue. The expression of the sodium iodide symporter is the key feature for specific iodine uptake. Patients with DTC have an excellent prognosis.

The therapeutic approach including surgery and remnant ablation with radioiodine should be risk-adapted to achieve an optimal treatment and to minimize treatment-related morbidity. Overtreatment should be avoided.

With regard to so-called low-risk carcinoma defined by the ATA there are controversial therapeutic approaches. The guidelines of the ATA recommend a lobectomy under certain conditions. Following the guidelines of the EANM a thyreodectomy with RIT should be performed (except PTC pT1a). However, long-term studies are currently not available. These studies are certainly necessary (against the background of the slow growth of the well-differentiated thyroid carcinoma) to decide which approach is appropriate. A risk-stratified follow-up is required since recurrences can occur over years. Furthermore, thyroid hormone substitution must be controlled.

The life span of most DTC patients does not differ from general population when appropriate treatment is given. The prognosis becomes poorer in patients with radioiodine refractory thyroid carcinoma. TKI have shown clinical effectiveness in iodine-refractory DTC with regard to progression free survival. A positive effect on overall survival could not be shown yet and has to be evaluated in further studies. However, therapy should be carried out in centers with special expertise.

In the current guidelines of the ATA and EANM there is no evidence-based treatment concept (or strong recommendation) for every situation. There are still open questions:

- The value of RIT under the condition of increasing serum level of Tg without a detectable correlatation in the morphological or functional imaging (i.e. iodine-negative whole-body scan);
- The benefit of a remnant ablation in patients with papillary microcarcinoma (very low risk of relapse, lymph node metastasis possible);
- Optimal activities of I-131 for safe and effective radioiodine ablation;
- The role of rhTSH as preparation for RIT to treat incomplete or non-resectable local recurrence or metastases;
- The role of a short LT4 withdrawal to reduce blood levels of iodine before RIT or diagnostic whole-body scan.

An analysis by the Cancer Genome Atlas Research Network identifies previously unknown genetic alterations and molecular subtypes of PTC. These alterations may lead to a more accurate diagnosis of tumors and potentially more targeted treatment [79]. Although in the current guidelines no explicit recommendation concerning the determination of molecular markers in the cyto-/histopathological specimen is made, further adjustments are to be expected in the future.

## Figures and Tables

**Table 1:** TNM Classification of thyroid cancer, 8th edition (modified from [12]).

TX	Primary Tumor Cannot be Assessed
T0	No evidence of primary tumor
T1	Tumor size maximum 2 cm, limited to the thyroid
T1a	Tumor size maximum 1 cm, limited to the thyroid
T1b	Tumor size >1 cm up to a maximum of 2 cm, limited to the thyroid
T2	Tumor size >2 cm up to 4 cm, limited to the thyroid
T3	Tumor size >4 cm, limited to the thyroid, or any tumor with macroscopic extrathyroidal extension (*Musculus sternohyoideus, Musculus sternothyreoideus, Musculus omohyoideus*)
T3a	Tumor size >4 cm, limited to the thyroid
T3b	Any tumor with macroscopic extrathyroidal extension (*M. sternohyoideus*, *M. sternothyreoideus, M. omohyoideus*)
T4a	Any tumor size with extrathyroidal extension beyond the thyroid capsule and invasion of subcutaneous soft tissue, larynx, trachea, esophagus and/or recurrent laryngeal nerve
T4b	Any tumor size with invasion of prevertebral fascia, mediastinal vessels or carotid artery
NX	Regional lymph nodes cannot be assessed
N0	No regional lymph node metastases
N1	Regional lymph node metastases
N1a	Lymph node metastases unilateral in level VI or upper mediastinum
N1b	Metastases in other unilateral, bilateral or contralateral cervical lymph nodes (level I, II, III, IV and V) or retropharyngeal
M0	No distant metastases
M1	Distant metastases

**Table 2:** Mutations of BRAF and TERTp in follicular-derived thyroid carcinoma and clinicopathological impact (modified from [15]).

Mutation	Histology	Clinicopathological Associations
BRAF	papillary thyroid carcinoma (PTC)	recurrence, multifocality, extrathyreoidal extension, lymph nodes metastasis, advanced stage, absence of capsule, vascular invasion, more aggressive histological subtype
BRAF	micro PTC	multifocality, extrathyreoidal extension, advanced stage, lymph node metastasis
BRAF	thyroid carcinoma derived from follicular cells	no association
TERT	papillary thyroid carcinoma	more advanced stage by tall cell variant, higher tumor size, vascular invasion, older age, poor outcome, lymph node and distant metastasis
TERT	thyroid carcinoma derived from follicular cells	more aggressive histologic variants, concomitant presence of mutated RAS/BRAF, age > 45, higher tumor size, vascular invasion, persistent or recurrent disease, lymph node metastasis

**Table 3:** WHO classification of papillary and follicular carcinoma of the thyroid (modified from [17]).

Histology	Histological Variants
Papillary carcinoma	Classic (usual) Clear cell variant Columnar cell variant Cribriform-morular variant Diffuse sclerosing variant Follicular variant Macrofollicular variant Microcarcinoma (occult, latent, small, microtumor) Oncocytic or oxyphilic variant (follicular/nonfollicular variant) Solid variant Tall cell variant Warthin-like variant
Follicular carcinoma	Clear cell variant Oncocytic (Hürthle cell) variant Mucinous variant With signet-ring cells

**Table 4:** Sonographic patterns and risk of malignancy (modified from [10]).

Ultrasound Features	Estimated Risk of Malignancy	Sonographic Pattern	FNA Size Cutoff
Solid hypoechogenic nodule or solid hypoechogenic component of a partially cystic nodule with one or more of the following features: irregular margins, microcalcification, taller rather than wide shape, rim calcifications with small extrusive soft tissue component, evidence of extrathyroidal extension (ETE)	>70–90%	Highly suspicious	>1 cm
Solid hypoechogenic nodule with smooth margins without microcalcification, taller rather than wide shape or signs of ETE	10–20%	Intermediate suspicion	>1 cm
Isoechogenic solid nodule or partally cystic nodule with eccentric solid areas without microcalcification, taller rather than wide shape or signs of ETE	5–10%	Low suspicion	>1.5 cm
Spongiform or partially cystic nodule without any of the sonographic features described above	<3%	Very low suspicion	>2 cm, alternative: observation without fine needle aspiration (FNA)
Purely cystic nodules without solid components	<1%	Benign	No biopsy

## Abbreviations

AKT1	RAC-α serine/threonine-protein kinase 1
ATA	American Thyroid Association
CT	computed tomography
DTC	differentiated thyroid carcinoma
EANM	European Association of Nuclear Medicine
ETE	extrathyroidal extension
ETA	European Thyroid Association
FAP	familial adenomatous polyposis
FNA	fine-needle aspiration biopsy
FTC	follicular thyroid carcinoma
FDG-PET	F-18-fluorodeoxy-glucose positron emission tomography
LT4	levothyroxine
MEN2	multiple endocrine neoplasia type 2
PAX8	paired box gene 8
PDTC	poorly differentiated thyroid carcinoma
PIK3CA	phosphatidylinositol 4,5-bisphosphate 3-kinase catalytic subunit α
PPARγ	peroxisome proliferator-activated receptor gamma
PTC	papillary thyroid carcinoma
PTEN	phosphatase and tensin homolog
PTMC	papillary microcarcinoma
RET	RET proto-oncogene
rhTSH	recombinant thyrotropin
RIT	radioiodine therapy
SPECT	single photon emission computed tomography
TERT	telomerase reverse transcriptase
Tg	serum thyroglobulin
TKI	receptor tyrosine kinase inhibitors
TP53	tumor protein p53
TSH	thyroid-stimulating hormone (also known as thyrotropin)
VEGF	vascular endothelial growth factor
WHO	World Health Organization
